# Effect of High Myopia on Dynamic Changes of Anterior Angle After Pharmacologic Mydriasis in Cataract Patients: A SS-ASOCT Study

**DOI:** 10.1167/tvst.10.6.25

**Published:** 2021-05-20

**Authors:** Wei Wang, Jiaqing Zhang, Xiaoxun Gu, Xuhua Tan, Xiaoting Ruan, Guangyao Yang, Xiaoyun Chen, Guangming Jin, Lanhua Wang, Ye Dai, Zhenzhen Liu, Lixia Luo, Yizhi Liu

**Affiliations:** 1 State Key Laboratory of Ophthalmology, Zhongshan Ophthalmic Center, Sun Yat-sen University, Guangzhou, Guangdong, China

**Keywords:** mydriasis, cataract, anterior segment optical coherence tomography (AS-OCT), swept source, high myopia

## Abstract

**Purpose:**

The purpose of this study was to evaluate the effect of high myopia on anterior angle change after pharmacologic mydriasis in patients with cataract using swept-source anterior segment optical coherence tomography (SS-ASOCT).

**Methods:**

This prospective cross-sectional study continuously recruited patients with cataract aged 40 years and older during the period August 2019 to August 2020. The anterior segment parameters, including central corneal thickness (CCT), anterior chamber depth (ACD), angle opening distance (AOD), angle recess area (ARA), trabecular iris space area (TISA), trabecular-iris angle (TIA), angle to angle width (ATA), and anterior chamber volume (ACV), were obtained using SS-ASOCT at baseline and 30 minutes after mydriasis. Regression analyses were performed to identify the factors related to the relative change of AOD500 (ΔAOD500).

**Results:**

A total of 938 patients (938 eyes) were included. The AOD500 decreased from 0.46 ± 0.22 mm to 0.40 ± 0.19 mm, with percent ΔAOD500 of −13.59% ± 37.73% (*P* = 0.005). The patients with high myopia had a smaller reduction of anterior angle parameters, with a percent ΔAOD500 of −22.74% ± 58.09%% in non-high myopic eyes and −0.84% ± 45.47% in high myopic eyes (*P* < 0.001). The stepwise multivariate regression demonstrated that the smaller reduction of AOD500 were independently associated with younger age (coefficient = −2.11, 95% confidence interval [CI] = −2.59 to −1.64, *P* < 0.001), presence of high myopia (coefficient = 15.35, 95% CI = 3.63 to 27.07, *P* = 0.010), greater baseline TISA500 (coefficient = 60.78, 95% CI = 8.75 to 112.82, *P* = 0.022), and ATA (coefficient = 11.21, 95% CI = 4.53 to 17.89, *P* = 0.001).

**Conclusions:**

The anterior chamber angle decreased after pharmacologic mydriasis in these patients with cataract. Angle shallowing after pharmacologic mydriasis was significantly less pronounced in high myopic eyes than in non-high myopic eyes.

**Translational Relevance:**

These findings are informative for the relative less risk of angle-closure glaucoma in highly myopic eyes.

## Introduction

Over the last 50 years, the prevalence of myopia has increased dramatically in East and Southeast Asia, such as China, Singapore, Japan, and Korea.^1^ In cities in these regions, 80% to 90% of high school students have myopia, and 10% to 20% have high myopia.^2^ The prevalence of primary angle closure glaucoma (PACG) was expected to decrease with the higher prevalence of myopia, which is considered to be a protective factor for angle closure.^3^^,^^4^ However, simulation analysis of the Liwan Eye Study reported that the myopia had minimal effect on PACG prevalence.^5^ Recent studies, on the other hand, have highlighted that a significant proportion of patients with PACG have myopia, with approximately one-third of rural patients with PACG in rural China having myopia, and nearly 37% of patients with PACG in Malaysia having myopia.^6^^,^^7^ PACG can also occur in high myopic eyes, with 1.9% to 2.6% of patients with PACG are highly myopic.^8^^,^^9^ Therefore, studies on the influence of myopia on PACG was limited and achieved inconsistent results.^5^^,^^10^

Anterior angle assessment is essential for PACG diagnosis and prognosis prediction.^11^ Anterior segment optical coherence tomography (AS-OCT) enables the visualization of the entire anterior structure in a single image. Population-based studies have confirmed that AS-OCT parameters, such as angle opening distance (AOD), trabecular iris space area (TISA), anterior chamber width (ACW), and lens vault (LV) were important determinants for PACG.^12^^,^^13^ In addition, the dynamic parameters after physiological and pharmacologic mydriasis were identified as novel risk factors for PACG.^14^ A study of Singaporean found that nearly one-fourth of patients with PACG were myopic, and that these eyes had altered ocular biometry, such as longer vitreous chamber depth and axial length (AL).^10^ However, the AS-OCT parameters were not analyzed in the study. In addition, AS-OCT parameters were measured by manually or semi-automatic software, which has relative high variability.^15^ The introduction of swept-source anterior segment optical coherence tomography (SS-ASOCT) provides a new imaging modality of anterior segment evaluation with a higher speed to obtain data, greater scanning range, and deeper scanning depth.^16^

In clinical practice, pharmacologic mydriasis was regularly performed before cataract surgery, which poses substantial risk for acute angle closure crisis. It is of clinical significance to evaluate anatomic changes after mydriasis, which is helpful to identify people at high risk for acute angle closure crisis. However, the effect of high myopia on anterior segment anatomic change after pharmacologic mydriasis remains elusive.^17^^,^^18^ Therefore, this study was designed to analyze the effect of high myopia on anterior angle change after pharmacologic mydriasis in patients with cataract using the latest swept source SS-ASOCT.

## Patients and Methods

### Patients

This prospective cross-sectional study was conducted at the Zhongshan Ophthalmic Center, Sun Yat-sen University, Guangzhou, China. Patients with cataract aged 40 years and older were continuously recruited from the Department of Cataract during the period August 2019 to August 2020. Subjects with any evidence of the following conditions were excluded: (1) diagnosed with PACG; (2) ocular diseases affecting anterior structures, such as pseudoexfoliation, lens subluxation, and traumatic cataract; (3) a history of intraocular (IOP) surgery or laser iridoplasty; (4) corneal abnormalities that would affect imaging, such as leucoma, keratoconus, or corneal scar; (5) poor fixation leading to low image quality or inability to cooperate with examinations; (6) severe artifacts or segmentation errors of anterior images; and (7) failure to finish the related examinations in compliance with the study protocol. The study was approved by the Institutional Review Board of the Zhongshan Ophthalmic Center (2019KYPJ033). This study was performed in accordance with the Declaration of Helsinki, and all subjects had signed a written informed consent.

### Questionnaire and Ocular Examination

All subjects underwent an interview via questionnaire and a comprehensive ocular examination on the same day. The customized questionnaires included history of ocular and systemic diseases, history of laser and surgeries, and medications. The ocular examinations included naked visual acuity (NVA) and best corrected visual acuity (BCVA), anterior and posterior segments evaluation, noncontact IOP measurements, and endothelial cell density (ECD). The IOL Master 700 (Carl Zeiss Meditec AG, Jena, Germany) was used to obtain ocular biometric parameters including corneal diameter (CD), anterior chamber depth (ACD), lens thickness (LT), flat meridian (K1), steep meridian (K2), axial length (AL), and pupil diameter (PD).

### SS-ASOCT Imaging

Anterior segment imaging was performed before and after mydriasis with a commercial SS-ASOCT (CASIA-2; Tomey Corporation, Nagoya, Japan), which uses a swept source laser with a wavelength of 1310 nm at a velocity of 30,000 A-scan/second. The high-definition angle protocol (AS H+D mode, 2 B-scans each with 800 A-scans over 16 mm) was adopted and performed by an experienced doctor under dark conditions. The subjects were asked to sit and fixate on the external lights during the examination, so scanning was focused on the central cornea to obtain a clear cross-sectional image of the angles. The examiner adjusted the device as appropriate during the examination to acquire the best quality images. Images with severe artifacts were excluded, including motion artifacts, data loss due to blinking, and failure of automatic stratification. The horizontal SS-ASOCT from nasal to temporal quadrants were automatically quantified by built-in software.

**Figure 1.**
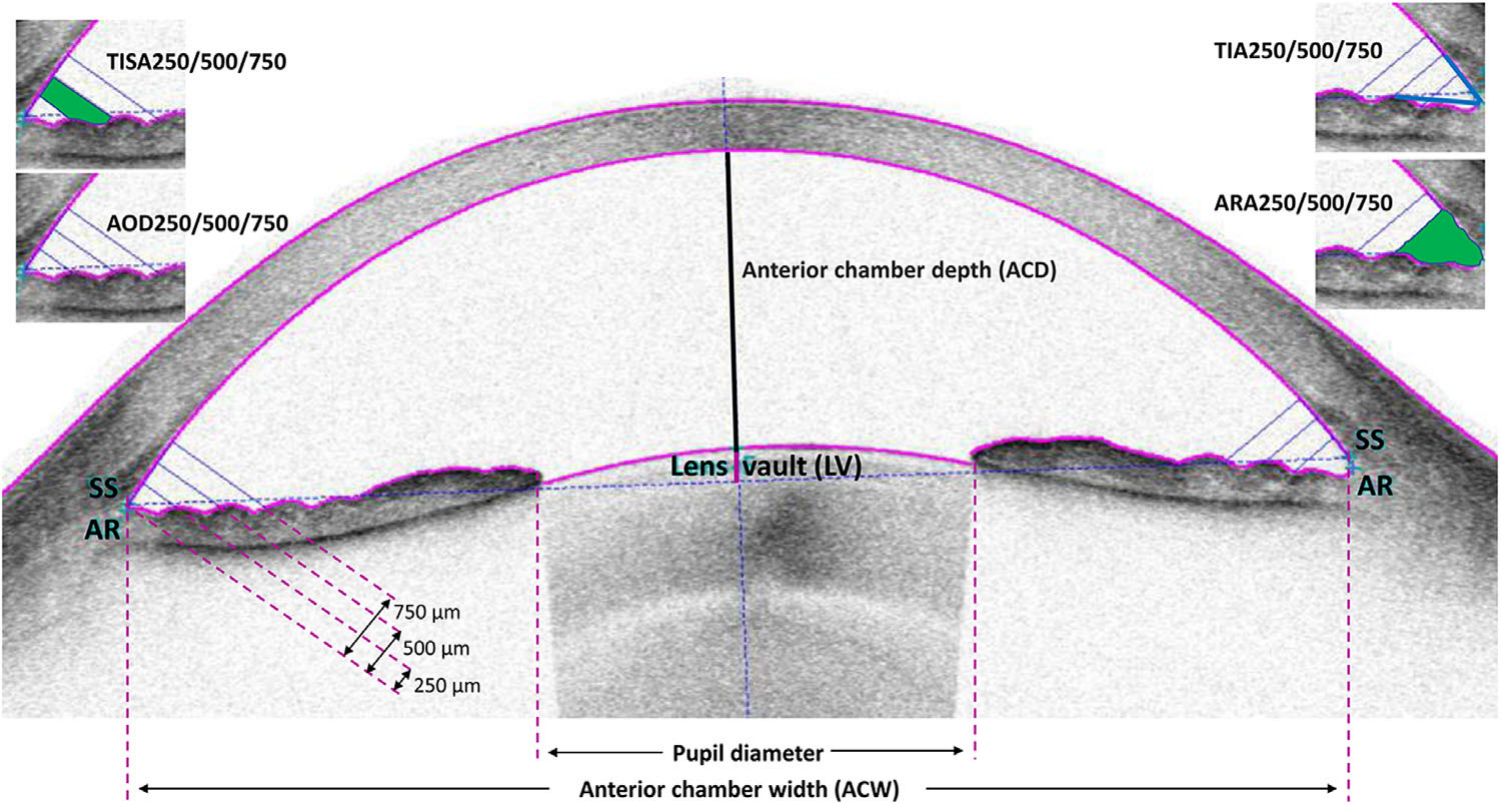
Illustration of parameters measured by swept-source anterior segment optical coherence tomography (SS-ASOCT). ACD = anterior chamber depth; ACW = anterior chamber width; AOD = angle opening distance; ARA = angle recess area; LV = lens vault; SS = scleral spur; TIA = trabecular iris angle; TISA = trabecular-iris space area; 250, 500, and 750 denote distance from SS in µm.

An independent author (J.Z.) reviewed all the SS-ASOCT images. The AS-OCT parameters were automatically measured by the built-in software. Manual adjustment was made in the event of software failure to accurately locate scleral spurs. Parameters (Fig. 1) including AOD, angle recess area (ARA), TISA, and trabecular-iris angle (TIA) were calculated at 25 µm, 500 µm, and 750 µm away from the scleral spur. AOD was defined as the distance from the iris surface at various distances from the scleral spurs perpendicular to a line drawn along the trabecular meshwork. ARA was defined as a triangular area, with the anterior line being the vertical line of AOD at various distances from the scleral spur with the outer line being the paries medialis of the corneosclera and the inner line being the iris anterior surface. TISA750 was defined as a trapezoid area by reducing from the ARA the area below a line from the scleral spur to the anterior iris perpendicular to the plane of the inner scleral wall. In addition, the central ACD, LV, ACW, and angle to angle width (ATA) were measured. ACW was defined as the horizontal distance between the bilateral scleral spurs. LV was defined as the distance from the anterior point of the lens perpendicular to a line drawn along the bilateral scleral spurs.

### Pharmacological Mydriasis

Pharmacological mydriasis was performed on all subjects after the first SS-ASOCT examination with the administration of 0.5% tropicamide plus 0.5% phenylephrine drops administered 3 times at 5 minute intervals. The second time examination of SS-ASOCT was performed at 30 minutes after the last drop when full dilation of the pupil was confirmed. It was considered full mydriasis when the pupil diameter was dilated larger than 6 mm and the light reflex disappeared. The subjects would be excluded if they could not follow the above standard procedures or meet the criteria of full mydriasis.

### Statistical Analysis

High myopia was defined as AL > 26.0 mm. The primary outcome of this study is the percent change of AOD500 (ΔAOD500) after mydriasis, which was calculated by AOD500 changes after mydriasis divided by AOD500 at baseline. All continuous variables were expressed using mean ± standard deviation. The Kolmogorov-Smirnov test was used to confirm the normal distribution. The paired *t*-test was used for comparing the differences before and after mydriasis. The student *t*-test was used to compare the mydriasis-induced anterior changes between high myopia and non-high myopic eyes. Univariate and stepwise multivariate linear regression analyses were used to identify the determinants of ΔAOD500, with ΔAOD500 as a dependent variable and clinical characteristics as independent variables, such as age, gender, AL, TISA500, ACD, and ACW. Variables that were significant at a level of < 0.20 in univariate analysis were included in multiple stepwise regression analysis to evaluate the factors independently correlated with ΔAOD500. All statistical analyses were performed using Stata MP 14.0 (StataCorp LP, College Station, TX, USA). A value of *P* < 0.05 was considered to have statistical significance unless otherwise specified.

## Results

### Demographic and Clinical Characteristics

A total of 938 patients (938 eyes) were included in the final analysis, with a mean age of 64.94 ± 8.91 years. Table 1 shows the demographic and clinical features of the subjects. The patients were 54.37% women and 45.63% men. The high myopic eyes had younger age, worse BCVA, higher AL, thicker central corneal thickness (CCT), flatter keratometry, greater CD, deeper ACD, and smaller LT (*P* < 0.05), whereas the distribution of age, systemic hypertension, diabetes mellitus, IOP, pupil diameter, and average and maximum nuclear density were similar between the groups (*P* > 0.05). For SS-ASOCT parameters at baseline, the high myopic eyes had significantly larger AOD, ARA, TISA, TIA, ACW, ATA, and smaller LV (Supplementary Table S1).

**Table 1.** Demographic and Clinical Characteristics of the Included Patients With Cataract

Characteristics	All	Non-High Myopia	High Myopia	*P* Value
No. of patients, %	938	818 (87.21%)	120 (12.79%)	–
**Demographic factors**				
Age, years	64.94 ± 8.91	65.33 ± 8.47	61.79 ± 10.99	**<0.001**
Female, %	54.37	54.65	52.50	0.659
Systemic hypertension, %	35.90	35.89	35.96	0.991
Diabetes mellitus, %	27.79	28.13	25.42	0.539
**Ocular factors before pupil dilation**				
BCVA, LogMAR unit	0.34 ± 0.37	0.31 ± 0.32	0.57 ± 0.56	**<0.001**
Intraocular pressure, mm Hg	15.5 ± 3.4	15.5 ± 3.3	15.9 ± 4.1	0.233
Corneal endothelial density, cells/mm^2^	2657.3 ± 367.4	2683.6 ± 316.6	2559.1 ± 510.9	0.125
Central corneal thickness, µm	544.9 ± 31.1	544.2 ± 31.0	551.3 ± 32.3	**0.039**
Anterior chamber depth, mm	2.75 ± 0.40	2.70 ± 0.38	3.01 ± 0.44	**<0.001**
Lens thickness, mm	4.65 ± 0.39	4.67 ± 0.37	4.53 ± 0.44	**<0.001**
Axial length, mm	23.79 ± 1.69	23.28 ± 0.75	27.09 ± 0.87	**<0.001**
Flat meridian (K1), D	43.82 ± 1.53	43.92 ± 1.50	43.17 ± 1.58	**<0.001**
Steep meridian (K2), D	44.62 ± 1.59	44.71 ± 1.55	44.02 ± 1.72	**<0.001**
Corneal diameter, mm	11.61 ± 0.40	11.59 ± 0.40	11.77 ± 0.41	**<0.001**
Pupil diameter, mm	4.09 ± 0.69	4.08 ± 0.75	4.11 ± 0.75	0.706
Average nuclear density	27.47 ± 5.21	27.36 ± 5.03	28.05 ± 5.99	0.550
Maximum nuclear density	87.99 ± 21.00	86.50 ± 20.06	90.74 ± 21.80	0.346

BCVA = best-corrected visual acuity; D = diopter; LogMAR= logarithm of the minimum angle of resolution.

Bold indicates statistical significance.

### Changes of the Anterior Segment Parameters After Mydriasis

Table 2 presents the changes of the anterior segment parameters before and after mydriasis. After mydriasis, parameters representing the anterior chamber dimension decreased significantly (all *P* < 0.001). The AOD500 decreased from 0.46 ± 0.22 mm to 0.40 ± 0.19 mm, with a mean percent change of -13.59% ± 37.73% (*P* = 0.005). The mean percent decrease of ARA500, TISA500, TIA500, and ACD were −11.47% ± 27.47%, −10.56% ± 41.70%, −9.18% ± 34.45%, and −4.60% ± 8.11%, respectively. The LV, ACW, and ATA did not change significantly (*P* > 0.05).

**Table 2.** Changes of Ocular Parameters After Pharmacologic Mydriasis in all Patients With Cataract

Parameter	Before Mydriasis	After Mydriasis	Absolute Difference	*P* Value	Percent Difference	*P* Value
IOP, mm Hg	15.54 ± 3.44	16.35 ± 3.00	0.14 ± 1.88	0.057	0.87% ± 11.91%	**<0.001**
AL, mm	23.79 ± 1.69	23.64 ± 1.34	0.0001 ± 0.03	0.930	0.0004% ± 0.10%	0.856
Pupil diameter, mm	4.09 ± 0.69	7.45 ± 0.67	3.37 ± 0.79	**<0.001**	82.44% ± 34.56%	**<0.001**
**AS-OCT parameters**						
AOD250, mm	0.33 ± 0.14	0.29 ± 0.12	−0.04 ± 0.11	**<0.001**	−11.86% ± 38.24%	**<0.001**
AOD500, mm	0.46 ± 0.22	0.40 ± 0.19	−0.06 ± 0.16	**<0.001**	−13.59% ± 37.73%	**0.005**
AOD750, mm	0.65 ± 0.30	0.54 ± 0.26	−0.11 ± 0.23	**<0.001**	−16.72% ± 32.64%	**<0.001**
ARA250, mm^2^	0.13 ± 0.07	0.11 ± 0.05	−0.02 ± 0.06	**<0.001**	−12.41% ± 25.32%	**<0.001**
ARA500, mm^2^	0.23 ± 0.11	0.20 ± 0.08	−0.03 ± 0.08	**<0.001**	−11.47% ± 27.47%	**0.006**
ARA750, mm^2^	0.37 ± 0.17	0.32 ± 0.13	−0.05 ± 0.13	**<0.001**	−13.23% ± 31.34%	**<0.001**
TISA250, mm^2^	0.08 ± 0.04	0.07 ± 0.03	−0.01 ± 0.03	**<0.001**	−9.52% ± 11.63%	**0.040**
TISA500, mm^2^	0.18 ± 0.07	0.16 ± 0.06	−0.02 ± 0.06	**<0.001**	−10.56% ± 41.70%	**0.003**
TISA750, mm^2^	0.33 ± 0.14	0.29 ± 0.12	−0.04 ± 0.10	**<0.001**	−12.39% ± 30.14%	**<0.001**
TIA250, degree	37.44 ± 15.28	33.89 ± 16.58	−3.55 ± 11.08	**<0.001**	−9.49% ± 33.26%	**<0.001**
TIA500, degree	32.55 ± 12.67	29.55 ± 13.89	−2.99 ± 9.63	**<0.001**	−9.18% ± 34.45%	**<0.001**
TIA750, degree	33.25 ± 12.72	28.83 ± 12.43	−4.42 ± 10.02	**<0.001**	−13.29% ± 26.39%	**<0.001**
ACD, mm	2.76 ± 0.40	2.63 ± 0.38	−0.13 ± 0.23	**<0.001**	−4.60% ± 8.11%	**<0.001**
LV, mm	0.23 ± 0.33	0.34 ± 0.33	0.12 ± 0.24	**<0.001**	52.58% ± 32.11%	0.335
ACW, mm	11.48 ± 0.43	11.47 ± 0.44	−0.01 ± 0.24	0.058	−0.09% ± 2.07%	0.461
ATA, mm	11.68 ± 0.55	11.67 ± 0.55	−0.01 ± 0.27	0.290	−0.09% ± 2.28%	0.215

IOP = intraocular pressure; AL = axial length; AOD = angle open distance; ARA = angle recess area; TISA = trabecular-iris space area; TIA = trabecular-iris angle; ACD = central anterior chamber depth; LV = lens vault; ACW = anterior chamber width; ATA = angle to angle width.

Bold indicates statistical significance.

### Comparisons of Mydriasis-Induced Changes in High and Non-High Myopic Eyes

**Figure 2.**
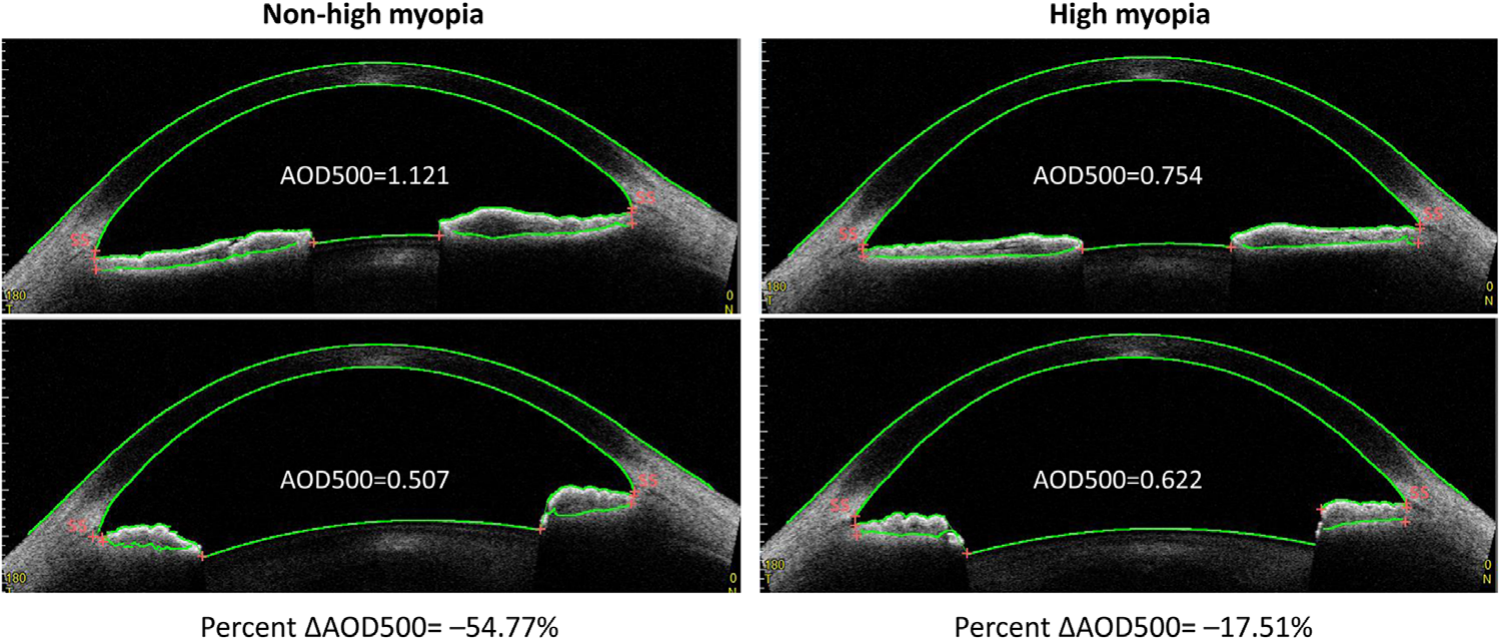
Percent changes of angle opening distance before and after pharmacologic mydriasis in high myopia and non-high myopia patients. AOD = angle opening distance; 500 denote distance from scleral spur in µm.

Table 3 presents the subgroup analyses comparing dynamic differences of the anterior segment between high myopic and non-high myopic eyes. The percent ΔAOD500 were −22.74% ± 58.09% in non-high myopic eyes and −0.84% ± 45.47% in high myopic eyes (*P* < 0.001). Similarly, the reduction of AOD250, AOD750, ARA750, TISA500, TISA750, TIA500, and TIA750 in high myopic eyes was significantly smaller than that of non-high myopic eyes (all *P* < 0.05).Figure 2 illustrates the reduction of AOD500 after pharmacologic mydriasis in a high myopic eye and non-high myopic eye.

**Table 3.** Comparisons of Mydriasis-Induced Changes Between High Myopia (Axial Length > 26 mm) and Non-High Myopia Eyes

	Absolute Changes (mm^2^/Degree/mm)			Percent Changes (%)		
Parameters	Non-High Myopia	High Myopia	*P* Value	Non-High Myopia	High Myopia	*P* Value
ΔIOP, mm Hg	0.14 ± 1.87	0.08 ± 1.98	**0.783**	1.58% ± 11.92%	0.78% ± 11.81%	**0.570**
ΔPupil diameter, mm	3.33 ± 0.77	3.63 ± 0.94	**0.003**	86.02% ± 32.81%	93.43% ± 45.79%	**0.081**
**AS-OCT parameters**						
ΔAOD250	−0.04 ± 0.09	−0.03 ± 0.15	0.142	−12.95% ± 38.10%	−1.64% ± 32.67%	**0.041**
ΔAOD500	−0.07 ± 0.15	−0.04 ± 0.18	**0.048**	−22.74% ± 58.09%	−0.84% ± 45.47%	**<0.001**
ΔAOD750	−0.12 ± 0.09	−0.10 ± 0.06	**0.026**	−14.09% ± 31.18%	−6.74% ± 32.17%	**0.023**
ΔARA250	−0.02 ± 0.05	−0.02 ± 0.07	0.833	−0.04% ± 65.01%	1.51% ± 44.71%	0.812
ΔARA500	−0.03 ± 0.07	−0.02 ± 0.10	0.567	−4.41% ± 18.95%	−0.48% ± 17.24%	0.424
ΔARA750	−0.06 ± 0.12	−0.04 ± 0.15	0.334	−8.86% ± 19.07%	−2.61% ± 14.08%	**0.005**
ΔTISA250	−0.01 ± 0.03	−0.01 ± 0.04	0.935	−2.53% ± 53.51%	−0.84% ± 39.29%	0.753
ΔTISA500	−0.03 ± 0.02	−0.01 ± 0.01	**0.018**	−6.00% ± 22.51%	−0.93% ± 24.98%	**0.038**
ΔTISA750	−0.05 ± 0.01	−0.03 ± 0.003	**0.015**	−9.73% ± 28.79%	−2.85% ± 22.41%	**0.023**
ΔTIA250	−4.10 ± 10.88	−2.13 ± 10.78	0.081	−8.78% ± 33.77%	−5.61% ± 27.36%	0.356
ΔTIA500	−3.70 ± 9.15	−1.06 ± 10.68	**0.006**	−8.91% ± 24.98%	−2.90% ± 17.98%	**0.048**
ΔTIA750	−5.12 ± 9.74	−2.87 ± 9.96	**0.026**	−13.88% ± 24.94%	−6.52% ± 26.71%	**0.005**
ΔACD	−0.13 ± 0.22	−0.13 ± 0.26	0.993	−4.54% ± 8.15%	−4.07% ± 7.67%	0.575
ΔLV	0.13 ± 0.24	0.10 ± 0.22	0.279	−21.71% ± 57.08%	−85.16% ± 72.54%	0.473
ΔACW	−0.04 ± 0.24	−0.05 ± 0.25	0.713	−0.31% ± 2.09%	−0.37% ± 2.09%	0.758
ΔATA	−0.01 ± 0.27	−0.005 ± 0.26	0.905	−0.04% ± 2.30%	−0.01% ± 2.20%	0.898

Δ = anterior segment changes after dilation (mydriasis minus baseline); IOP = intraocular pressure; AOD = angle open distance; ARA = angle recess area; TISA = trabecular-iris space area; TIA = trabecular-iris angle; ACD = central anterior chamber depth; LV = lens vault; ACW = anterior chamber width; ATA = angle to angle width.

Bold indicates statistical significance.

### General and Ocular Factors Associated With ΔAOD500

Table 4 shows the univariate and multivariate regression results. The age, hypertension, and LT were negatively associated with values of ΔAOD500, whereas the CD, presence of high myopia, TISA500, and ATA were positively associated with values of ΔAOD500 in univariate regression. The multiple stepwise regression analysis showed that the greater ΔAOD500 value (namely smaller narrowing of angle) were independently associated with younger age (coefficient = −2.11, 95% CI, −2.59 to −1.64, *P* < 0.001), presence of high myopia (coefficient = 15.35, 95% CI = 3.63 to 27.07, *P* = 0.010), greater baseline TISA500 (coefficient = 60.78, 95% CI = 8.75 to 112.82, *P* = 0.022), and larger baseline ATA (coefficient = 11.21, 95% CI = 4.53 to 17.89, *P* = 0.001). Further adjusting for the changes of pupil diameter, the results of multivariable regression achieved consistent results (Supplementary Table S2).

**Table 4.** Univariate and Stepwise Multivariate Linear Regression of Percent ΔAOD500 With the General and Ocular Parameters

	Univariate Analysis		Stepwise Multivariate Analysis	
Parameters	Coefficient (95% CI)	*P* Value	Coefficient (95% CI)	*P* Value
**Demographic parameters**				
Age, per 1-year increase	−2.58 (−2.89 to −2.26)	**<0.001**	−2.11 (−2.59 to −1.64)	**<0.001**
Sex, male versus female	5.07 (−1.45 to 11.59)	0.127		
Systemic hypertension, yes versus no	−10.25 (−18.14 to −2.37)	**0.011**		
Diabetes mellitus, yes versus no	−5.56 (−12.86 to 1.73)	0.135		
**Ocular parameters before pupil dilation**				
Presence of high myopia (AL > 26.0 mm)	21.90 (12.35 to 31.45)	**<0.001**	15.35 (3.63 to 27.07)	**0.010**
Anterior chamber depth, per 1-mm increase	4.69 (−3.50 to 12.88)	0.261		
Lens thickness, per 1-mm increase	−41.89 (−50.46 to −33.32)	**<0.001**		
Corneal diameter, per 1-mm increase	8.81 (0.74 to 16.87)	**0.032**		
Flat meridian (K1), per 1-D increase	−1.68 (−3.77 to 0.41)	0.115		
Flat meridian (K2), per 1-D increase	−1.34 (−3.35 to 0.66)	0.189		
TISA500, per 1-mm^2^ increase	66.02 (23.76 to 108.29)	**0.002**	60.78 (8.85 to 112.82)	**0.022**
LV, per 1-mm increase	−4.31 (−14.21 to 5.58)	0.393		
ACW, per 1-mm increase	0.47 (−6.97 to 7.92)	0.901		
ATA, per 1-mm increase	13.95 (8.14 to 19.76)	**<0.001**	11.21 (4.53 to 17.89)	**0.001**
Average nuclear density, per 1-unit increase	−0.38 (−1.92 to 1.16)	0.627		
Maximum nuclear density, per 1-unit increase	0.10 (−0.29 to 0.49)	0.616		
**Ocular changes after mydriasis**				
ΔIOP, per 1-mm Hg increase	−0.16 (−1.88 to 1.55)	0.853		
ΔPupil diameter, per 1-mm increase	−3.60 (−7.88 to 0.69)	0.100		

Δ = anterior segment changes after dilation (mydriasis minus baseline); IOP = intraocular pressure; AOD = angle open distance; TISA = trabecular-iris space area; LV = lens vault; ACW = anterior chamber width; ATA = angle to angle width.

Bold indicates statistical significance.

## Discussion

The effect of high myopia on mydriasis-induced anterior angle change have not been elucidated.^11^^,^^18^ This study used the latest SS-ASOCT to quantify the anterior segment change before and after pharmacologic mydriasis in Chinese patients with cataract who had the highest risk for PACG. The main findings were as follows: (1) the anterior chamber angle decreased after mydriasis in this population; (2) those with high myopia had less angle narrowing after mydriasis; and (3) age, axial length, TISA500, and ATA at baseline were important factors that influenced dynamic change of the anterior segment after mydriasis.

This study demonstrated that pupil dilation with tropicamide and phenylephrine results in the narrowing of the drainage angle, which is consistent with previous studies on anatomic changes induced by pharmacologic dilation.^17^^–^^21^ It was reported that in healthy eyes, the iris volume decreases and the angle width stays the same, decreases, or increases with pharmacologic dilation, and in angle closure eyes or the fellow eyes of patients with unilateral acute angle closure AOD500 and TISA500 decrease substantially.^17^^–^^23^ Pharmacological mydriasis was associated with the risk of causing acute angle events. However, the angle closure may occur when the pharmacologic effect is wearing off and the pupil is midway through returning to normal, combined with the posterior rotation of the ciliary body returning to a more anterior position. This study found a decrease of AOD suggesting an increase of pupil block force following pupillary dilation in cataract eyes.

Epidemic myopia poses substantial challenges for East Asians. Myopia and long axial length were reported to be associated with a lower risk for PACG in population-based studies.^3^^–^^5^ Spherical equivalent was commonly used to define high myopia in previous studies. A study of Singaporean patients with PACG did not observe any difference in anterior segment parameters (e.g. ACD, corneal curvature, lens thickness, and LV) between myopic and hyperopic eyes, but 3 of the 11 highly myopic patients with PACG in that study had AL even < 22 mm.^10^ Another study of Malaysian patients with PACG detected no difference between myopic and hyperopic eyes in AL (22.95 ± 0.98 vs. 22.73 ± 0.92 mm).^7^ These imply that axial myopia and lenticular myopia were confused in the aforementioned studies. PACG occurs usually in people over 40 years of age, and thus the definition of high myopia by refractive status in patients with PACG may be misleading, because the high myopia might arise from an increase in the refractive index of the lens with age rather than from axial growth of the eye.^24^ Therefore, the high myopia was defined based on AL rather than refractive status, which presents a strength of this study.

Previous studies on anterior segment parameters in myopic eyes showed conflicting results in different studies. Li et al.^24^ reported that angle closure eyes with longer axial length had flatter cornea and larger ACW. However, Yong et al.^10^ demonstrated that myopia had little influence on ACD in Singaporean patients. Chong et al.^25^ also reported that the anterior segment in myopic eyes with narrow angles were not different from open angles. As shown in this current study (Supplementary Table S1), the presence of high myopia displayed greater anterior chamber and smaller LV, which might suggest a lower risk of angle-closure glaucoma in high myopic eyes. Further longitudinal studies are warranted to clarify the impact of myopia on AS-OCT parameters.

The dynamic changes of anterior segment parameters captured by AS-OCT were identified as an important risk for PACG, however, only a small number of studies are available and the results are controversial. The Handan Eye Study in rural China demonstrated that dynamic iris changes after mydriasis were not correlated with SE or AL in multivariate regression models.^21^ A population-based study on Japanese patients with cataract also found that ΔAOD500 after mydriasis was not correlated with AL.^17^ However, Malyugin et al.^26^ reported that high myopic and non-high myopic eyes had different ACD changes in response to mydriasis in a Russian population. Aptel et al.^18^ reported that the AL was significantly correlated with dynamic change of iris volume/pupil after pharmacologic dilation in France. The discrepancy may be related to the different AS-OCT instruments, ethnic variations, and urban-rural differences. This study found that high myopia was a powerful prediction factor for dynamic change of the anterior segment. Angle shallowing after mydriasis was significantly less pronounced in eyes with high myopia than in non-high myopic eyes. Thus, the findings supported the notion that high myopia is a protective factor for PACG.

The latest SS-ASOCT enabled a fully automatic quantitative evaluation of the parameters. To the best of our knowledge, this was the first study to use the latest SS-ASOCT to investigate the impact of mydriasis on the anterior segment in Chinese patients with cataract. However, the limitations of this study should be noted. First, this is a hospital-based study, which might introduce selection bias. Second, the cross-sectional nature prevents the causal inference. Further prospective studies with long-term follow-up are needed to validate the clinical value of our findings. Third, gonioscopy was not performed. It has been reported that angle synechia might interfere with results measured by AS-OCT.^27^^,^^28^ Therefore, the generalization of the results to patients with PACG warrants further study.^29^ Finally, only Chinese patients were included in the study, and the studies in other ethnic populations are needed to confirm or refute the findings.^4^^,^^7^

In summary, the anterior chamber angle decreased after pharmacologic mydriasis in these patients with cataract. Angle shallowing after pharmacologic mydriasis was significantly less pronounced in high myopic eyes than in non-high myopic eyes. Further longitudinal studies of various ethnicity are warranted to clarify the impact of myopia on AS-OCT parameters.

## Supplementary Material

Supplement 1
