# Supplementary material for: Effect of High Myopia on Dynamic Changes of Anterior Angle After Pharmacologic Mydriasis in Cataract Patients: A SS-ASOCT Study
Source: Transl Vis Sci Technol. 2021 May 20;10(6):25. doi: 10.1167/tvst.10.6.25 (PMC8142703; doi:10.1167/tvst.10.6.25)
Supplement: Supplement 1 [file tvst-10-6-25_s001.pdf]

Supplementary Table 1. Baseline distribution of SS-ASOCT parameters in non-high myopic and high myopic eyes.

Parameter	All		Non-High myopia		High myopia		P-value
	Mean	SD	Mean	SD	Mean	SD	
AOD250, mm	0.33	0.14	0.32	0.13	0.41	0.17	**<0.001**
AOD500, mm	0.46	0.22	0.44	0.21	0.59	0.23	**<0.001**
AOD750, mm	0.65	0.30	0.62	0.29	0.84	0.32	**<0.001**
ARA250, mm^2	0.13	0.07	0.13	0.07	0.15	0.08	**0.001**
ARA500, mm^2	0.23	0.11	0.22	0.10	0.28	0.12	**<0.001**
ARA750, mm^2	0.37	0.17	0.36	0.17	0.46	0.19	**<0.001**
TISA250, mm^2	0.08	0.04	0.08	0.03	0.09	0.04	**<0.001**
TISA500, mm^2	0.18	0.07	0.18	0.07	0.22	0.09	**<0.001**
TISA750, mm^2	0.33	0.14	0.31	0.13	0.41	0.16	**<0.001**
TIA250, degree	37.44	15.28	36.28	14.87	42.12	15.98	**<0.001**
TIA500, degree	32.55	12.67	31.09	11.71	39.69	13.85	**<0.001**
TIA750, degree	33.25	12.72	31.92	12.26	40.47	12.00	**<0.001**
LV, mm	0.23	0.33	0.25	0.32	0.05	0.29	**<0.001**
ACW, mm	11.48	0.43	11.45	0.43	11.61	0.45	**<0.001**
ATA, mm	11.68	0.55	11.66	0.56	11.83	0.52	**0.003**

AOD=angle open distance; ARA=angle recess area; TISA=trabecular-iris space area; TIA= trabecular-iris angle; ACD= central anterior chamber depth; LV=lens vault; ACW=anterior chamber width; ATA=angle to angle width.

Bold indicates statistical significance.

Supplementary Table 2. Multivariate linear regression of percent ΔAOD500 after adjusting each other factors and changes of pupil diameter after pupil dilation.

Parameter	Coefficient (95%CI)	P-value
Age, per 1-year increase	-2.74 (-3.12, -2.35)	**<0.001**
Presence of high myopia (AL>26.0 mm)	15.09 (5.51, 24.68)	**0.002**
TISA500, per 1-mm^2 increase	74.36 (31.85, 116.88,)	**0.001**
ATA, per 1-mm increase	10.96 (5.37, 16.56)	**<0.001**
ΔPupil diameter, per 1-mm increase	-1.42 (-5.29, 2.45)	0.472

AL=Axial length; TISA=trabecular-iris space area; ATA=Angle to angle width.

Bold indicates statistical significance.
